# P-2081. Prototype Assay for Detection of Carbapenem-Resistant Acinetobacter baumannii Complex (CRABC)

**DOI:** 10.1093/ofid/ofae631.2237

**Published:** 2025-01-29

**Authors:** Brian Lee, Natacha Martins Sorenson, Hai Nguyen, Arrash Moghaddasi, Aishwarya Sathish, Patrick Lin, Kyle C Cady

**Affiliations:** Roche Diagnostics Solutions, Pleasanton, California; Roche Diagnostics, Santa Clara, California; Roche Diagnostics, Santa Clara, California; Roche Diagnostics, Santa Clara, California; Roche Diagnostics, Santa Clara, California; Roche Diagnostics, Santa Clara, California; Roche Diagnostics, Santa Clara, California

## Abstract

**Background:**

Carbapenem-resistant *Acinetobacter baumannii* complex (CRABC) is recognized as an urgent/critical antimicrobial resistance (AMR) threat, causing hospital-acquired and ventilator-associated pneumonia (HAP/VAP) and bacteremia. Due to limited treatment options, there is an urgent need for novel targeted antibiotics against CRABC. As these drugs become available, their timely and appropriate use will require the rapid identification of patients with CRABC infection. We evaluated the performance of a prototype assay for the rapid detection of CRABC directly from bronchoalveolar lavage (BAL), sputum/endotracheal aspirate (S/ETA), and positive blood culture (PBC) samples.

**Methods:**

A prototype assay was designed for the qualitative detection of *A. baumannii* complex (ABC) harboring the most prevalent associated carbapenemase genes (*bla*OXA-23*, bla*OXA-24*, bla*OXA-58, and/or *bla*NDM). The prototype assay was developed for use on the high-throughput **cobas®** 5800/6800/8800 Systems with a defined oligo pool and positive control. A total of 452 BAL, 681 S/ETA, and 392 PBC samples (including prospective, archived, and contrived specimens) were tested by the prototype assay and compared with routine microbiology for ABC detection and the Unyvero Hospitalized Pneumonia or BCU panel for carbapenemase detection. Discrepant resolution was performed using the BioFire® FilmArray® Pneumonia or BCID Panel and/or the Streck ARM-D® Kit, OXA assay, depending on the type of discrepancy.

**Results:**

After resolution of discrepant results, the overall percentage agreement, positive percent agreement, and negative percent agreement between the prototype assay and the comparator methods (culture/nucleic acid amplification tests [NAATs]) was 97.1%, 98.5%, and 96.5%, respectively, for BAL; 99.3%, 100%, and 99.1%, respectively, for S/ETA; and 99.2%, 100%, and 99.1%, respectively, for PBC.

**Conclusion:**

This prototype assay exhibited a high percentage agreement for the detection of CRABC in BAL, S/ETA, and PBC samples when compared to culture/NAATs. A future assay based on this prototype could be evaluated in a clinical trial to establish its utility in facilitating timely and appropriate antibiotic selection for patients with HAP/VAP or bacteremia caused by CRABC.

**Disclosures:**

Brian Lee, MD, GE Healthcare: Stocks/Bonds (Public Company)|Invitae: Stocks/Bonds (Public Company)|Merck: Stocks/Bonds (Public Company)|OPKO Health: Stocks/Bonds (Public Company)|Roche Diagnostics Solutions: Employment|Roche Diagnostics Solutions: Stocks/Bonds (Public Company)|Spero Therapeutics: Stocks/Bonds (Public Company) Natacha Martins Sorenson, PhD, Roche Diagnostics Solutions: Employment|Roche Diagnostics Solutions: Stocks/Bonds (Public Company) Hai Nguyen, n/a, Roche Diagnostics Solutions: Employment Arrash Moghaddasi, n/a, Roche Diagnostics Solutions: Employment Aishwarya Sathish, n/a, Roche Diagnostics Solutions: Employment Patrick Lin, n/a, Roche Diagnostics Solutions: Employment Kyle C. Cady, PhD, Roche Diagnostics Solutions: Employment|Roche Diagnostics Solutions: Stocks/Bonds (Public Company)

